# Effectiveness of clear aligners in achieving proclination and intrusion of incisors among Class II division 2 patients: a multivariate analysis

**DOI:** 10.1186/s40510-023-00463-6

**Published:** 2023-04-03

**Authors:** Xinyu Yan, Xiaoqi Zhang, Linghuan Ren, Yi Yang, Qingxuan Wang, Yanzi Gao, Qingsong Jiang, Fan Jian, Hu Long, Wenli Lai

**Affiliations:** Department of Orthodontics, State Key Laboratory of Oral Diseases and National Clinical Center for Oral Research, West China Hospital of Stomatology, Sichuan University, No. 14, Section 3, Ren Min Nan Road, Chengdu, 610041 China

**Keywords:** Clear aligner, Class II division 2, Tooth movement, Multivariate linear regression

## Abstract

**Background:**

The predictability of incisor movement achieved by clear aligners among Class II division 2 patients is poorly understood. The aim of this retrospective study was to determine the effectiveness of clear aligners in proclining and intruding upper incisors and its influencing factors.

**Methods:**

Eligible patients with Class II division 2 malocclusion were included. For clear aligner therapy, three types of incisor movements were designed: proclination, intrusion and labial movement. Pre-treatment and post-treatment dental models were superimposed. The differences between predicted and actual (DPA) tooth movement of incisors were analyzed. Univariate and multivariate linear regression were used to analyze the potential influencing factors.

**Results:**

A total of 51 patients and their 173 upper incisors were included. Actual incisor proclination and intrusion were less than predicted ones (both *P* < 0.001), while actual labial movement was greater than predicted one (*P* < 0.001). Predictability of incisor proclination and intrusion was 69.8% and 53.3%, respectively. Multivariate linear regression revealed that DPA of proclination was significantly positively associated with predicted proclination (*B* = 0.174, *P* < 0.001), ipsilateral premolar extraction (*B* = 2.773, *P* < 0.001) and ipsilateral canine proclination (*B* = 1.811, *P* < 0.05), while negatively associated with molar distalization (*B* = − 2.085, *P* < 0.05). The DPA of intrusion was significantly positively correlated with predicted intrusion (*B* = 0.556, *P* < 0.001) while negatively associated with labial mini-implants (*B* = − 1.466, *P* < 0.001). The DPA of labial movement was significantly positively associated with predicted labial movement (*B* = 0.481, *P* < 0.001), while negatively correlated with molar distalization (*B* = − 1.004, *P* < 0.001), labial mini-implants (*B* = − 0.738, *P* < 0.001) and age (*B* = − 0.486, *P* < 0.05).

**Conclusions:**

For Class II division 2 patients, predicted incisor proclination (69.8%) and intrusion (53.3%) are partially achieved with clear aligner therapy. Excessive labial movement (0.7 mm) of incisors may be achieved. Incisor movement is influenced by predicted movement amount, premolar extraction, canine proclination, molar distalization, mini-implants and age.

## Background

Class II division 2 malocclusion is distinguishable from Class II division 1 by clinical manifestations of lingually inclined upper incisors and anterior deepbite, with a relatively low incidence [1, 2]. Upper incisors are often excessively extruded, which may result in abnormal tooth attrition, gingival trauma, or esthetic compromises [2, 3]. Thus, orthodontic treatment for this malocclusion includes three types of incisor movement, i.e., proclination, intrusion and labial movement. Proclination and intrusion are pivotal to correction of lingually inclined and extruded incisors, but too much labial movement may lead to labial bone defects.

Clear aligners generate tooth movement depending on the force released by elastic deformation and are increasingly widely used for the advantages of esthetics, comfort and cleaning convenience [4, 5]. Moreover, treatment goal visualization can be achieved using digital technique brought by clear aligners, which allows more accurate three-dimensional control of tooth movement. However, a large body of evidence reveals that difference exists between predicted and actual tooth movements, and that the predictability of tooth movement through clear aligners varies among different types of tooth movement, ranging from 30 to 88% [6]. Although it has been demonstrated that clear aligner is able to manage Class II division 2 patients [7], the predictability of incisor movement has been poorly understood.

Accumulated evidence has demonstrated that the effectiveness of proclination and intrusion of incisors are affected by a variety of factors related to patients, e.g., age, gender, tooth crown morphology and root length [8–10]. In terms of clear aligners, materials, aligner thickness, attachment designs and production techniques have impacts on incisor movements [11–15].

To date, the clinical effectiveness of clear aligner therapy in managing Class II division 2 malocclusion has been largely unknown. Therefore, we conducted this study to examine the effectiveness of clear aligner therapy in treating lingually inclined upper incisors among Class II division 2 patients and its potential influencing factors, which may offer clinical guidelines for practitioners in treatment planning and prognosis evaluation.

## Material and methods

### Study participants

This retrospective study was conducted based on patients receiving orthodontic treatment from January 2018 to December 2021 in Department of Orthodontics, West China Hospital of Stomatology, Chengdu, China. The protocol for this study was approved by Ethics Committee of West China Hospital of Stomatology (WCHSIRB-D-2021-547). Inclusion criteria were (1) patients diagnosed with Class II division 2 malocclusion according to cephalometric and model analysis (U1-SN < 103°, deepbite and Class II molar relationship); (2) subjects treated using Invisalign (Align Technology, USA) clear aligner therapy; (3) permanent dentition; (4) completing the first series of aligners without midcourse correction. Exclusion criteria were as follows: (1) anterior teeth with abnormal morphology; (2) severe caries or periapical diseases; (3) moderate to severe periodontitis; (4) diseases that influenced bone metabolism; (5) incomplete pre- or post-treatment data.

### Clear aligner therapy

Prior to treatment, intra-oral scanning and radiographic examinations of patients were prescribed. Thus, digital dentitions and cone-beam computed tomography (CBCT) images were obtained. For clear aligner therapy, proclination and intrusion of upper incisors were designed to obtain a normal overbite and overjet. Moreover, premolar extraction or upper molar distalization was executed to correct molar relationship. Orthodontic mini-implants and orthodontic elastics were prescribed when necessary. All the participants were asked to wear clear aligners for at least 22 h per day and to change aligners every 10 days.

### Data collection

The following demographic information and clinical treatment data were obtained: age, sex, U1-SN (the angle between sella-nasion plane and axis of upper central incisor), the number of aligner staging, teeth (central or lateral incisors) that would be proclined (crown movement ≥ 5°) and intruded (crown movement ≥ 0.5 mm), premolar extraction, molar distalization, staging design (simultaneous or separate design of incisor proclination and molar distalization), the type of incisor attachments (Power Ridge, optimized attachments, or none), the type of attachments on ipsilateral canines (vertical rectangle, horizontal rectangle, optimized attachments, or none), ipsilateral canine proclination and intrusion, labial mini-implants, and Class II elastic traction. Labial mini-implants were prescribed if impinging overbite was present, and orthodontic Class II elastics were used if molar distalization was designed following incisor proclination. Specifically, labial mini-implants were placed between upper central incisors or between central and lateral incisors, and orthodontic elastics were worn from the palatal precision cuts of clear aligners to the labial mini-implants. Class II elastics were worn from the labial precision cuts of canines to the buccal buttons on mandibular first molars.

**Fig. 1:**
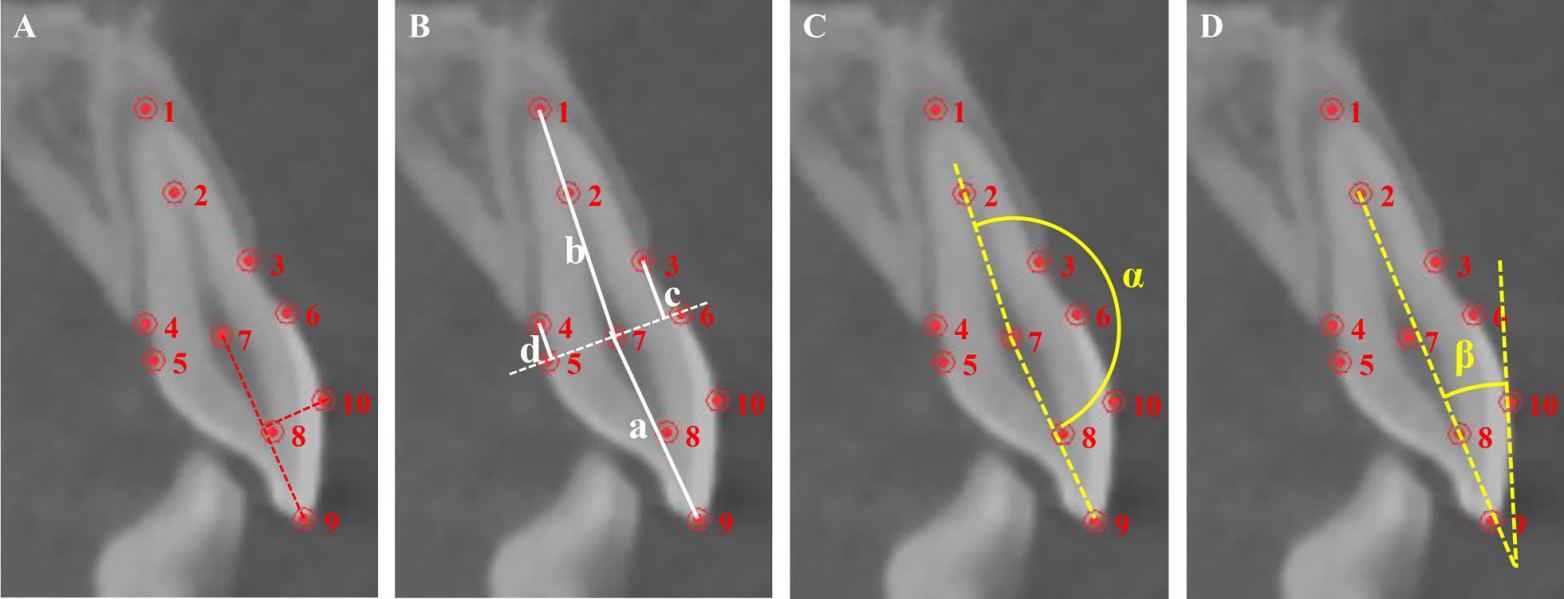
Measurement of dental anatomic characteristics. **A** Point identification (1-root apex; 2-point of lower 1/3 root; 3-labial alveolar ridge crest; 4-palatal alveolar ridge crest; 5-palatal cemental-enamel junction (CEJ); 6-labial CEJ; 7-midpoint of labial and palatal CEJ; 8-apex of crown pulp cavity; 9-incisal edge point; 10-projection of midpoint of crown axis on labial surface). **B** Line distance measurement (a-crown length; b-root length; c-height of labial alveolar bone; d-height of palatal alveolar bone). **C** Crown-root angle; **D** Labial surface-axis angle

The dental anatomic characteristics were acquired by importing pre-treatment CBCT into Mimics Medical 21.0 (Materialise, Belgium). Line distances and angles were measured on the median sagittal section of each tooth, including crown and root length, height of labial and lingual alveolar bone, crown-root angle and labial surface-axis angle (Fig. 1). Specifically, crown-root angle was the angle between crown axis and root axis, which was defined as positive when extension of root axis was positioned labially to crown axis. Labial surface-axis angle was defined as the angle between long axis and the tangent line of labial crown surface, indicating convexity of the labial surface of crown. All the measurement was conducted by the same operator for three times with an interval of 10 days, and the average values were calculated.

### Model superimposition and measurement

**Fig. 2:**
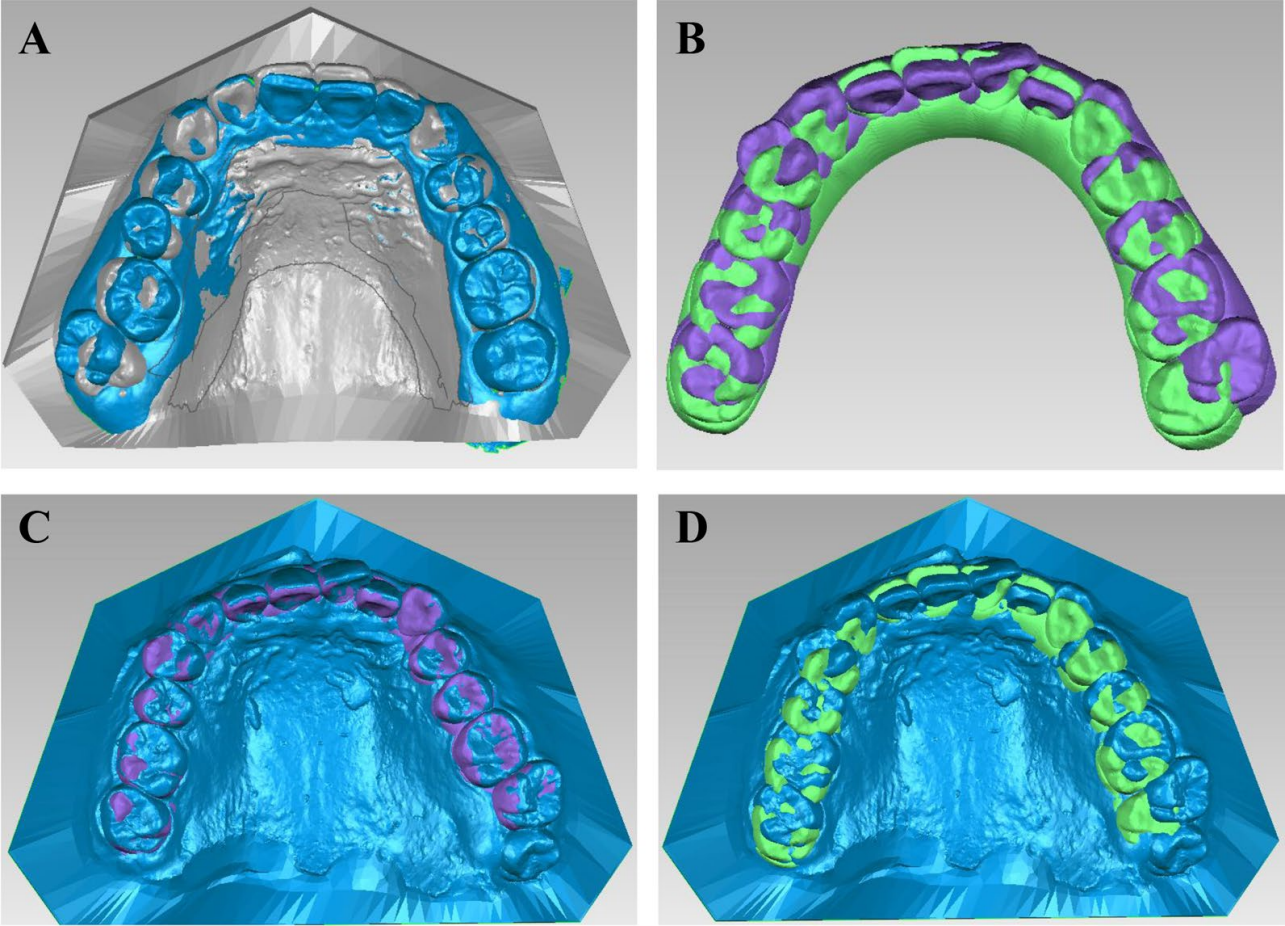
Superimposition of models. **A** Superimposition of T0 (blue) and T1 (gray) based on the palatal vault region. **B** Automatic registration of T0-CC (purple) and T1-CC (green) when exported from ClinCheck. **C** Superimposition of T0 (blue) and T0-CC (purple) by the same dental arches **D** Superimposition of T0 (blue) and T1-CC (green) mediated by T0-CC

Predicted and actual pre- and post-treatment digitized models were, respectively, superimposed. Specifically speaking, actual pre- and post-treatment digitized models (T0 and T1) were acquired through intraoral scanning using iTero (Align Technology, USA). Predicted pre- and post-treatment digitized models (T0-CC and T1-CC) simulated in the treatment plan were obtained from ClinCheck program (Align Technology, USA). Digitized models were saved as stereolithography (STL) files and imported into Geomagic Studio 2014 (Raindrop Technology Limited, USA). T0 and T1 were superimposed based on the palatal vault region, which was proved as a relatively stable reference by previous studies [16] (Fig. 2A). T0-CC and T1-CC were automatically registered when exported from ClinCheck (Fig. 2B). T0 and T0-CC were superimposed by the same dental arches, and thereby superimposition of T0 and T1-CC was achieved (Fig. 2C, D).

**Fig. 3:**
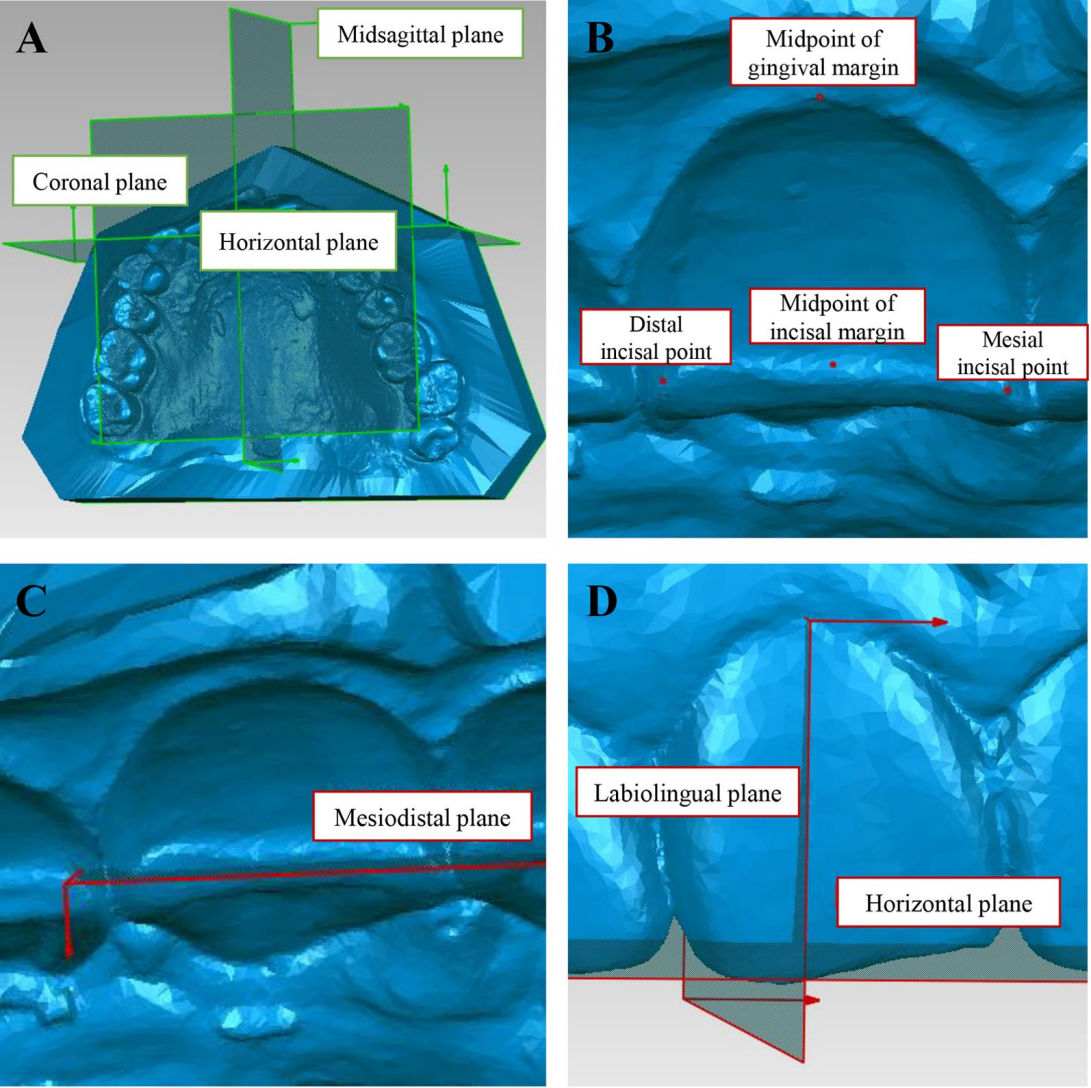
Construction of reference planes. **A** Three reference planes constructed on T0. **B** Point identification on upper incisors. **C** Mesiodistal plane constructed on upper incisors. **D** Labiolingual plane constructed on upper incisors

After superimposition of T0 and T1/T1-CC models, a three-dimensional coordinate system was established by three reference planes on T0. The horizontal plane was constructed by mesial cusps of bilateral maxillary first molars and mesial incisal point of right maxillary central incisor. Midsagittal plane was constructed through mid-palatal suture vertically to the horizontal plane. And the coronal plane was constructed through incisive papilla vertically to above two planes (Fig. 3A). Four points were identified on each upper incisor: mesial incisal point, distal incisal point, midpoint of incisal margin, and midpoint of gingival margin (Fig. 3B). Moreover, a local coordinate system was established on each tooth. Mesiodistal plane was constructed through mesial and distal incisal points vertically to the horizontal plane (Fig. 3C). Labiolingual plane was constructed through midpoint of gingival margin vertically to horizontal and mesiodistal planes (Fig. 3D). The landmarks on incisors of T0 were transferred to identical teeth of T1 and T1-CC through tooth crown surface superimposition, and all the local coordinate systems were constructed based on the horizontal plane of T0.

**Fig. 4:**
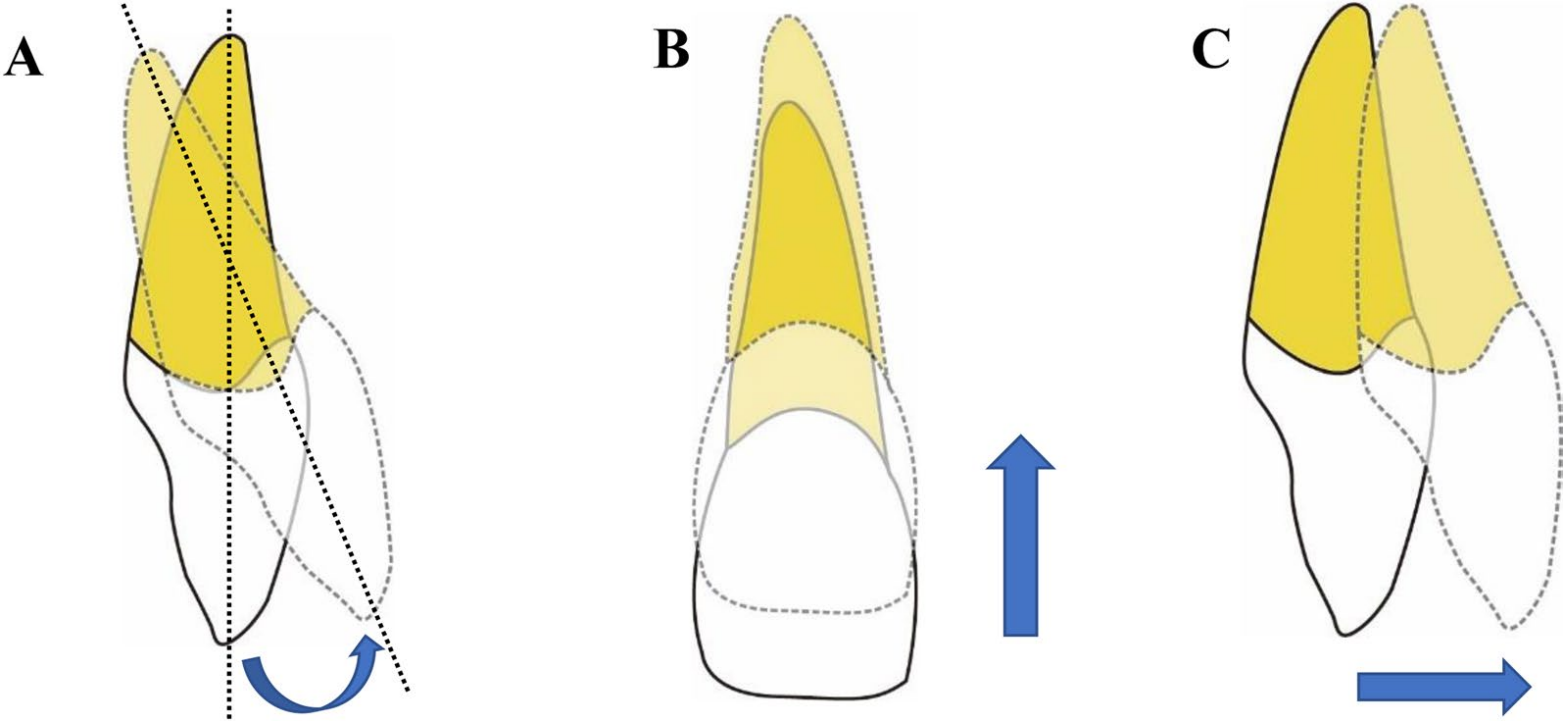
Schematic plots of three types of tooth movement. **A** Proclination. **B** Intrusion. **C** Labial movement

For upper incisors, three types of tooth movement were analyzed, i.e., proclination, intrusion and labial movement. Specifically, proclination was defined as labial inclination of crown axis, and intrusion and labial movement were, respectively, defined as the gingival and labial movement of the incisor edge (Fig. 4).

**Fig. 5:**
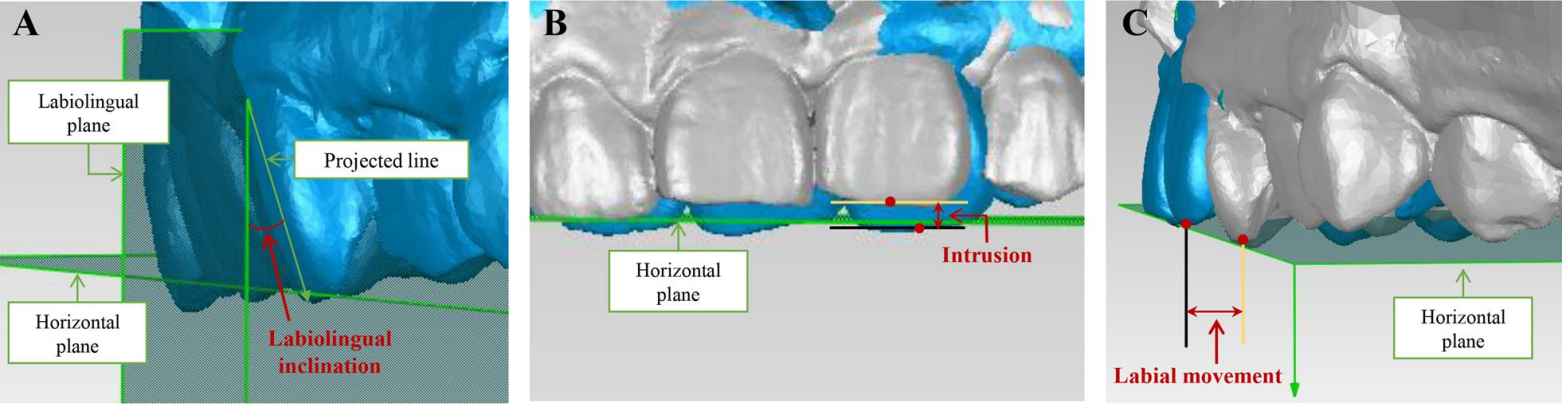
Measurement of tooth movement. **A** Measurement of incisor labiolingual inclination. **B** Measurement of incisor height. **C** Measurement of labial movement amount

For measurement of incisor labiolingual inclination, the crown axis connecting incisal midpoints and gingival margins was projected to labiolingual plane. The angle between the projected line and the line vertical to horizontal plane was defined as incisor labiolingual inclination (Fig. 5A). The amount of proclination was calculated as the difference value between pre- and post-treatment incisor labiolingual inclination. The distance between the lines parallel to the horizontal plane passing through midpoints of incisal margin of T0 and T1/T1-CC was measured as incisor intrusion amount (Fig. 5B). The distance between the lines vertical to the horizontal plane passing through midpoints of incisal margin of T0 and T1/T1-CC was measured as labial movement amount (Fig. 5C). The calculation formulas were as follows: differences between predicted and achieved tooth movement (DPA) = predicted tooth movement amount–actual tooth movement amount; the predictability of tooth movement = actual tooth movement amount/predicted tooth movement amount × 100%.

### Statistical analysis

Superimposition and measurement were repeated by the same operator after two weeks on 10% randomly selected models, and interclass correlation coefficient (ICC) was used to evaluate intraoperator agreement. Shapiro–Wilk test was used to evaluate data distribution. Actual and predicted tooth movement amount were compared using paired t test. Univariate and multivariate linear regression analyses were used to evaluate the independent associations between DPA and variables including demographic, clinical treatment and dental anatomic characteristics (variables with *P* < 0.2 in univariate analyses were included in multivariate analyses). All statistical analyses were conducted using IBM SPSS statistics 24.0 (SPSS, USA) and GraphPad Prism 7 (GraphPad Software Inc., USA), and a *P* < 0.05 was considered statistically significant.

## Results

### Characteristics of study participants

A total of 51 eligible participants were included. The majority of participants were adults (45, 88.2%) and females (38, 74.5%), with an average age of 25.1 years and U1-SN of 93.1°. The baseline clinical characteristics are listed in Table 1. Screened by designed tooth movement amount in ClinCheck, 173 upper incisors were included for analyses, among which 153 were adult tooth (88.4%), 127 were female tooth (73.4%), and 97 were central incisors (56.1%). Other clinical treatment and dental anatomic characteristics are listed in Table 2.

**Table 1:** Characteristics of study population

Characteristics	Value/Number
Age (years)	25.1 ± 6.7
Adult	45 (88.2%)
Female	38 (74.5%)
Ipsilateral premolar extraction	17 (33.3%)
Upper molar distalization	38 (74.5%)
Separate anterior and posterior tooth movement	26 (51.0%)
Labial mini-implants	6 (11.8%)
Class II elastics used during molar distalization	14 (27.5%)

**Table 2:** Characteristics of study upper incisors

Characteristics	Value/Number
Ipsilateral premolar extraction	57 (32.9%)
Upper molar distalization	99 (57.2%)
Separate anterior and posterior tooth movement	106 (61.3%)
Power Ridge or attachments on incisors	
Power Ridge	127 (73.8%)
Optimized attachment	24 (14.0%)
None	21 (12.2%)
Attachments on ipsilateral canines	
Vertical rectangular attachment	70 (43.8%)
Optimized attachment	43 (26.9%)
Horizontal rectangular attachment	24 (15.0%)
None	23 (14.4%)
Labial mini-implants	22 (12.7%)
Class II elastics used during molar distalization	50 (28.9%)
Dental anatomic characteristics	
Positive crown-root angle	103 (64.8%)
Absolute value of crown-root angle (°)	177.0 ± 2.9
Labial surface-axis angle (°)	20.6 ± 3.5
Crown-root ratio	0.9 ± 0.2
Height of labial alveolar bone (mm)	2.4 ± 2.3
Height of palatal alveolar bone (mm)	1.4 ± 0.8

### Comparison of actual and predicted tooth movement amount

ICCs for amount of proclination, intrusion and labial movement were, respectively, 0.994, 0.990 and 0.996, indicative of high intraoperator agreement of superimposition and measurement (Table 3). As shown in Table 4, significant differences were found between actual and predicted amounts of the three types of tooth movement (all *P* < 0.001). Specifically, the actual and predicted amount of incisor proclination was 9.7 ± 7.3 degrees and 13.9 ± 7.3 degrees, respectively. The DPA of incisor proclination was 4.2 ± 4.5 degrees. For incisor intrusion, the actual and predicted amount was, respectively, 0.8 ± 1.1 mm and 1.5 ± 1.1 mm, leading to a DPA of 0.7 ± 1.1 mm. Notably, the actual amount of labial movement of upper incisors was significantly greater than the predicted one (1.3 ± 1.5 mm vs. 0.6 ± 2.2 mm), resulting in a negative DPA of labial movement (− 0.7 ± 1.5 mm).

**Table 3:** Intraoperator agreement analysis

Measurement index	ICC	95%CI
Proclination amount	0.994	0.991, 0.996
Intrusion amount	0.990	0.984, 0.994
Labial movement amount	0.996	0.993, 0.998

**Table 4:** Comparison of actual and predicted tooth movement amount

Measurement index	Actual tooth movement	Predicted tooth movement	DPA	*P* value
Proclination amount (°)	9.7 ± 7.3	13.9 ± 7.3	4.2 ± 4.5	< 0.001*
Intrusion amount (mm)	0.8 ± 1.1	1.5 ± 1.1	0.7 ± 1.1	< 0.001*
Labial movement amount (mm)	1.3 ± 1.5	0.6 ± 2.2	− 0.7 ± 1.5	< 0.001*

*P < 0.05 indicated statistical significance

### Influencing factors for DPAs of different types of tooth movement

For incisor proclination, nine variables were associated with the DPA of proclination in univariate level (Table 5, *P* < 0.2), including predicted proclination, age, tooth, ipsilateral premolar extraction, upper molar distalization, staging design, ipsilateral canine proclination, attachments on incisors, and attachments on ipsilateral canines. While in the multivariate analysis, the number of significant factors reduced from nine to four (*P* < 0.05). DPA of incisor proclination was positively correlated with predicted proclination (*B* = 0.174, *P* < 0.001), ipsilateral premolar extraction (*B* = 2.773, *P* < 0.001), ipsilateral canine proclination (*B* = 1.811, *P* < 0.05), while negatively associated with ipsilateral molar distalization (*B* = − 2.085, *P* < 0.05).

**Table 5:** Univariate and multivariate linear regression analyses for DPA of upper incisor proclination

Variables	Univariate	Multivariate	
	*P* value	*B* (95%CI)	*P* value
*Predicted proclination*	< 0.001**	0.174 (0.087, 0.261)	< 0.001**
Absolute value of crown-root angle	0.406		
Labial surface-axis angle	0.581		
Crown-root ratio	0.414		
Height of labial alveolar bone	0.777		
Height of palatal alveolar bone	0.224		
Crown-root angle			
Minus	0.996		
Plus	Reference		
Age			
Adolescent	0.005**	− 0.855 (− 2.952, 1.242)	0.421
Adult	Reference		
Gender			
Male	0.657		
Female	Reference		
Tooth			
Lateral incisor	0.038**	− 1.051 (− 2.320, 0.218)	0.104
Central incisor	Reference		
*Ipsilateral premolar extraction*			
Yes	< 0.001**	2.773 (1.236, 4.311)	< 0.001**
No	Reference		
*Upper molar distalization*			
Yes	< 0.001**	− 2.085 (− 4.062, − 0.109)	0.039**
No	Reference		
Separate anterior and posterior movement			
Yes	0.045**	− 0.650 (− 2.541, 1.241)	0.498
No	Reference		
*Ipsilateral canine proclination*			
Yes	0.010**	1.811 (0.204, 3.419)	0.027**
No	Reference		
Labial mini-implants			
Yes	0.902		
No	Reference		
Class II elastics			
Yes	0.631		
No	Reference		
Attachments on incisors			
Power Ridge	0.039**	1.045 (− 0.873, 2.963)	0.283
Optimized attachment	0.495	0.883 (− 1.533, 3.299)	0.471
None	Reference		
Attachments on ipsilateral canines			
Vertical rectangular attachment	0.010**	0.862 (− 1.224, 2.948)	0.415
Optimized attachment	0.042**	0.491 (− 1.685, 2.667)	0.656
Horizontal rectangular attachment	0.467	0.127 (− 2.391, 2.645)	0.921
None	Reference		

^*^P < 0.2 indicated the variable could be included in multivariate analysis; **P < 0.05 indicated statistical significance

As displayed in Table 6, predicted intrusion, absolute value of crown-root angle, crown-root ratio, age, tooth, ipsilateral canine intrusion, labial mini-implants, and attachments on ipsilateral canines were associated with the DPA of intrusion using univariate tests (*P* < 0.2). By multivariate linear regression analysis, only predicted amount of intrusion and labial mini-implants were independent influencing factors for DPA of intrusion (*P* < 0.05). Specifically, predicted amount of intrusion was positively correlated with DPA of intrusion (*B* = 0.556, *P* < 0.001). DPA of intrusion was significantly smaller when labial mini-implants were used compared to the opposite (*B* = − 1.466, *P* < 0.001).

**Table 6:** Univariate and multivariate linear regression analyses for DPA of upper incisor intrusion

Variables	Univariate	Multivariate	
	*P* value	*B* (95%CI)	*P* value
*Predicted intrusion*	< 0.001**	0.556 (0.424, 0.688)	< 0.001**
Absolute value of crown-root angle	0.152*	0.013 (− 0.034, 0.060)	0.596
Labial surface-axis angle	0.307		
Crown-root ratio	0.155*	− 0.632 (− 1.377, 0.113)	0.096
Height of labial alveolar bone	0.245		
Height of palatal alveolar bone	0.228		
Crown-root angle			
Minus	0.303		
Plus	Reference		
Age			
Adolescent	0.080*	− 0.077 (− 0.565, 0.412)	0.757
Adult	Reference		
Gender			
Male	0.249		
Female	Reference		
Tooth			
Lateral incisor	0.113*	− 0.080 (− 0.380, 0.220)	0.597
Central incisor	Reference		
Ipsilateral premolar extraction			
Yes	0.299		
No	Reference		
Ipsilateral canine intrusion			
Yes	0.002**	0.181 (− 0.105, 0.466)	0.212
No	Reference		
*Labial mini-implants*			
Yes	< 0.001**	− 1.466 (− 1.850, − 1.083)	< 0.001**
No	Reference		
Class II elastics			
Yes	0.460		
No	Reference		
Attachments on incisors			
Power Ridge	0.295		
Optimized attachment	0.891		
None	Reference		
Attachments on ipsilateral canines			
Vertical rectangular attachment	0.079*	0.327 (− 0.123, 0.777)	0.153
Optimized attachment	0.098*	0.189 (− 0.296, 0.675)	0.442
Horizontal rectangular attachment	0.539	0.200 (− 0.307, 0.707)	0.436
None	Reference		

^*^P < 0.2 indicated the variable could be included in multivariate analysis; **P < 0.05 indicated statistical significance

In univariate linear regression analysis, ten variables were associated with the DPA of labial movement of upper incisors (*P* < 0.2), including predicted amount of labial movement, absolute value of crown-root angle, labial surface-axis angle, height of labial alveolar bone, age, gender, tooth position, upper molar distalization, labial mini-implants, and attachment on incisors (Table 7). In multivariate linear regression analysis, four independent influencing factors for DPA of labial movement were identified (*P* < 0.05), among which the most significant was predicted amount of labial movement (*β* = 0.712). Predicted amount of labial movement was positively related to DPA of labial movement (*B* = 0.481, *P* < 0.001). And the DPA of labial movement was significantly decreased when ipsilateral upper molars were designed distalized (*B* = − 1.004, *P* < 0.001) or labial mini-implants were used (*B* = − 0.738, *P* < 0.001) compared to the opposite, or in adolescents compared to adults (*B* = − 0.486, *P* < 0.05).

**Table 7:** Univariate and multivariate linear regression analyses for DPA of upper incisor labial movement

Variables	Univariate	Multivariate	
	P value	B (95%CI)	P value
*Predicted labial movement*	< 0.001**	0.481 (0.410, 0.551)	< 0.001**
Absolute value of crown-root angle	0.002**	− 0.011 (− 0.059, 0.036)	0.640
Labial surface-axis angle	0.003**	− 0.015 (− 0.053, 0.024)	0.461
Crown-root ratio	0.280		
Height of labial alveolar bone	0.027**	− 0.037 (− 0.097, 0.023)	0.222
Height of palatal alveolar bone	0.252		
Crown-root angle			
Minus	0.376		
Plus	Reference		
*Age*			
Adolescent	0.060*	− 0.486 (− 0.957, − 0.015)	0.043**
Adult	Reference		
Gender			
Male	< 0.001**	0.011 (− 0.318, 0.339)	0.949
Female	Reference		
Tooth			
Lateral incisor	0.185*	0.178 (− 0.091, 0.447)	0.193
Central incisor	Reference		
Ipsilateral premolar extraction			
Yes	0.402		
No	Reference		
*Upper molar distalization*			
Yes	0.002**	− 1.004 (− 1.265, − 0.743)	< 0.001**
No	Reference		
Separate anterior and posterior movement			
Yes	0.287		
No	Reference		
Ipsilateral canine proclination			
Yes	0.838		
No	Reference		
*Labial mini-implants*			
Yes	0.008**	− 0.738 (− 1.113, − 0.363)	< 0.001**
No	Reference		
Class II elastics			
Yes	0.357		
No	Reference		
Attachments on incisors			
Power Ridge	0.667	0.180 (− 0.224, 0.584)	0.380
Optimized attachment	0.091*	0.398 (− 0.124, 0.920)	0.134
None	Reference		
Attachments on ipsilateral canines			
Vertical rectangular attachment	0.311		
Optimized attachment	0.480		
Horizontal rectangular attachment	0.209		
None	Reference		

*P < 0.2 indicated the variable could be included in multivariate analysis; **P < 0.05 indicated statistical significance

## Discussion

The key points for treatment of Class II division 2 are to correct the inclination of incisors and open the bite through proclining and intruding upper incisors, while avoiding excessive labial movement of incisors to prevent labial bone defects. As an esthetic and comfortable orthodontic appliance, clear aligners are applied in more and more complex cases of malocclusion, including Class II division 2. However, one of the most critical problems of clear aligners is the limited predictability of tooth movement, especially for the control of labiolingual tipping of incisors. Hence, it is of great importance to improve the efficiency of tooth movement achieved through clear aligners.

Our study demonstrated that actual incisor proclination (9.7 degrees) and intrusion (0.8 mm) were significantly smaller than the predicted ones (proclination: 13.6 degrees; intrusion: 1.5 mm). Thus, the predictability of incisor proclination and intrusion was 69.8% and 53.3%, respectively. This finding was slightly different from those published previously where the predictability of incisor proclination ranged from 37.6 to 64.5% [13, 14, 17], and the achievement of upper incisor intrusion ranged from 32.5 to 51.2% [14, 18, 19], which could be explained by the fact that different types of malocclusions (not limited to Class II division 2 cases) were included in other studies. Particularly, it has been revealed in one study that the predictability of incisor proclination was 100% [20]. This high predictability of incisor proclination was due to the fact that only limited tooth movement (within the initial 12 stages) was evaluated. But generally speaking, the consistent conclusion is that realization of upper incisor movement is limited in clear aligner therapy, indicating that overtreatment planning is necessary.

Noteworthily, the average actual labial movement amount was greater than predicted in this study, indicating that unwanted excessive labial movement of upper incisors occurred in most of cases as a side effect. Combined with limited proclination and intrusion, this is consistent with a previous study on non-extraction cases treated with clear aligners. This study showed that the actual final position of central incisors was more labial, occlusal and lingually inclined than designed [21]. It is proposed that the correction of lingual inclination of upper incisors by clear aligners was actually more dependent on labial movement of crowns instead of controlled lingual movement of roots as designed, thus leading to excessive labial movement of upper incisors. Indeed, difficulty in root control of clear aligners has been reported in plenty of previous studies. It has been demonstrated that movement of anterior teeth achieved by clear aligners was mainly tipping movement even if root-controlling movement was initially designed, which is demonstrated by a larger movement amount of crowns than that of roots [22, 23]. A biomechanical study also confirmed that clear aligners tended to lift up during torque movement, and thus no effective force couples could be generated for further root control [24].

The multivariate linear regression analyses demonstrated that the DPA of proclination of upper incisors was independently associated with predicted amount of proclination, ipsilateral premolar extraction, ipsilateral upper molar distalization, and ipsilateral canine proclination. The more the DPA of proclination was, the larger the discrepancy between predicted and actual proclination was, thus indicating more need for overtreatment of tooth proclination. Predicted amount of proclination was positively related to the DPA of proclination (*B* = 0.174, *P* < 0.001), indicating that increasing predicted amount for overtreatment might facilitate actual proclination, while the efficiency might not be necessarily improved. 2.085° of DPA of proclination was decreased when the ipsilateral maxillary molars were designed distalized compared to the opposite (*B* = − 2.085, *P* < 0.05), which is because increased labial force on anterior teeth as a counterforce of molar distalization. Under the same magnitude of force, the movement amount of posterior teeth would be less than that of anterior teeth due to different areas of periodontal membranes, hence it should be noted that realization of molar distalization might be insufficient when it is performed simultaneously with anterior proclination [25]. Compared with non-extraction of ipsilateral tooth, 2.773° of DPA of proclination was increased when ipsilateral tooth was extracted (*B* = 2.773, *P* < 0.001), which might be explained by three reasons. Firstly, the labial force exerted by aligners in anterior region cannot be effectively expressed due to stress-breaking effect resulting from extraction space; secondly, more torque loss might occur during long-distance retraction of anterior teeth to close extraction space; thirdly, labial force on upper incisors was relatively lacking because no molar distalization would be designed in extraction cases. Moreover, 1.811° of DPA of proclination was increased when the ipsilateral canine was designed proclined (*B* = 1.811, *P* < 0.05), which is on account of more stable anchorage provided by the ipsilateral canine when it was designed to non-procline or even lingually inclined.

Our study reported that predicted amount of intrusion and labial mini-implants are two independent influencing factors for DPA of intrusion of upper incisors. The more the DPA of intrusion was, the larger the discrepancy between predicted and actual intrusion was, thus indicating more need for overtreatment of tooth intrusion. Specifically, predicted amount of intrusion was positively related to DPA of intrusion (*B* = 0.556, *P* < 0.001), which is consistent with the influential effect of predicted amount of proclination on DPA of proclination. 1.466 mm of DPA of intrusion was decreased when labial mini-implants were used, revealing significant intrusive effect of labial mini-implants combined with elastics crossing the incisal edge of upper incisors.

The independent influencing factors for DPA of labial movement of upper incisors are predicted amount of labial movement, ipsilateral upper molar distalization, age, and labial mini-implants. Since the DPA values of labial movement were on average negative, the less the DPA of labial movement was, the more unwanted labial movement occurred. Consistent with above findings, predicted amount of labial movement was positively correlated with DPA of labial movement (*B* = 0.481, *P* < 0.001). 1.004 mm of DPA of labial movement was decreased when ipsilateral upper molars were designed distalized compared to the opposite, which is due to labial counterforce on upper incisors generated by molar distalization as abovementioned. When other covariates were adjusted, 0.486 mm of DPA of labial movement was decreased in adolescents compared with adults (*B* = − 0.486, *P* < 0.05), which might be explained by more sensitive tissue reactivity and active bone remodeling that leads to easier tooth movement. The effect of age on orthodontic tooth movement has been confirmed by previous animal studies, in which tooth movement amount of younger individuals was significantly larger than that of older counterparts, and the velocity of tooth movement declined with increased age [26, 27]. 0.738 mm of DPA of labial movement was decreased when labial mini-implants were used (*B* = − 0.738, *P* < 0.001), indicating apparent labial force could be generated by anterior implants combined with elastics in addition to intrusive effect. A three-dimensional finite element study demonstrated anterior implants combined with elastics crossing the incisal edge of upper incisors to the lingual side of aligners could effectively increase incisor intrusion and root lingual torque [28]. Nevertheless, based on our results that actual amount of labial movement was larger than predicted, it can be inferred that excessive unwanted labial movement of upper incisors occurred in the cases using labial mini-implants, with simultaneous mesial movement of molars.

This study evaluated the potential influencing factors for the treatment of Class II division 2 malocclusion using clear aligners for the first time. As a retrospective study, confounding effects of plenty of variables cannot be eliminated. To investigate the associations of multiple variables with one dependent factor, simply using several univariate analyses tends to produce statistically correct but clinically wrong results [29]. It is widely acknowledged that combining the results of both univariate and multivariate analyses may improve the accuracy and authenticity of the retrospective clinical studies that aim to reveal the independent effects of variables with confounding effects adjusted [30–32]. The limitation of the study is the relatively small sample size, although it has met the requirement relative to the number of studied variables. Therefore, the sample size should be further enlarged to improve the reliability of multivariate regression analyses, and more covariates could be taken into consideration with a larger sample size. Besides, this observational study cannot imply certain causal relationships, but it is more likely to provide preliminary evidence for further randomized controlled trials (RCTs) to testify the possibly significant influencing factors.

## Conclusions

**Fig. 6:**
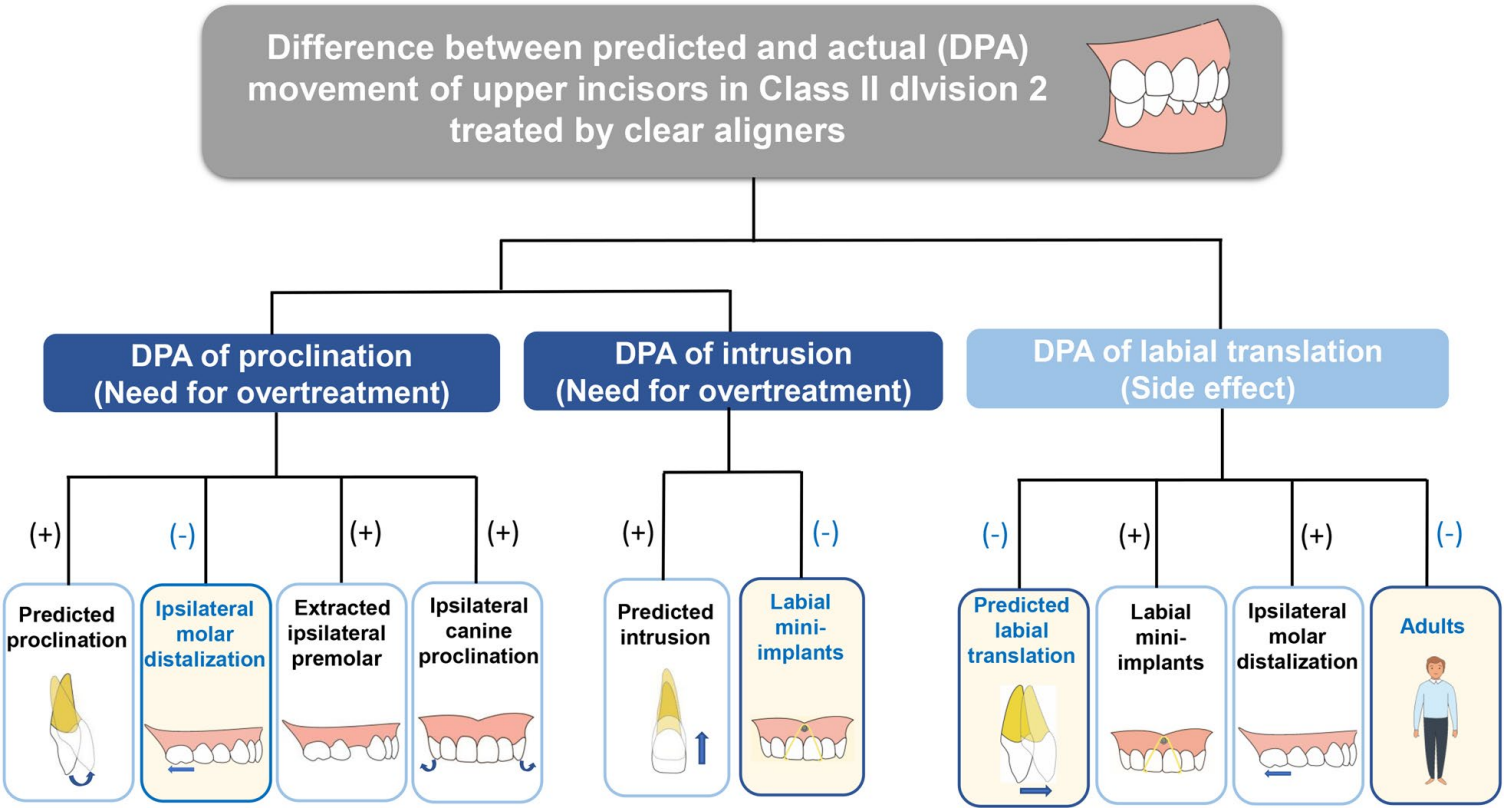
The influencing factors for effectiveness of tooth movement in Class II division 2 cases

1. Clear aligner therapy is able to partly achieve incisor proclination (69.8%) and intrusion (53.3%), while excessive labial movement of incisors occurred as a side effect that should be prevented.
2. The DPA of incisor proclination is increased when more predicted proclination amount, extraction of ipsilateral premolar, non-distalization of ipsilateral molar or proclination of ipsilateral canine is designed, indicating more need for overtreatment of proclination.
3. The DPA of incisor intrusion is increased with more predicted intrusion amount or without labial mini-implants, thus needing overtreatment of intrusion.
4. Excessive labial movement could be prevented when less amount of predicted labial movement, non-distalization of ipsilateral molar or no labial mini-implants is designed. Adolescents are more susceptible to excessive labial movement of incisors (Fig. 6).

## Data Availability

The datasets used and/or analyzed during the current study are available from the corresponding author on reasonable request.
